# Extrauterine Growth Restriction: Definitions and Predictability of Outcomes in a Cohort of Very Low Birth Weight Infants or Preterm Neonates

**DOI:** 10.3390/nu12051224

**Published:** 2020-04-26

**Authors:** Chiara Peila, Elena Spada, Francesca Giuliani, Giulia Maiocco, Melissa Raia, Francesco Cresi, Enrico Bertino, Alessandra Coscia

**Affiliations:** 1 Neonatal Unit, Department of Public Health and Pediatric, University of Turin. Via Ventimiglia III, 10126 Turin, Italy; 2 Department of Otorhinolaryngology-Head and Neck Surgery, University of Eastern Piedmont, 28100 Novara, Italy

**Keywords:** extrauterine growth restriction, postnatal growth of preterm neonates, EUGR definition, EUGR and auxological outcomes

## Abstract

Extrauterine Growth Restriction (EUGR) refers to inadequate growth during hospitalization. Current definitions for EUGR are varied and can be classified as cross-sectional (weight at a given t-time <10th centile) or longitudinal (weight loss between birth and a given t-time >1SD). Different t-times are also considered in literature, such as 36 weeks of gestational age (GA) or age at discharge. The aim of this study is to investigate whether EUGR could predict the auxological outcome at 24–30 months, and to evaluate the agreement between cross-sectional and longitudinal definitions. In total, 1589 infants with GA <30 weeks or birthweight ≤ 1500 g and without major congenital anomalies were included in this study. Cross-sectional and longitudinal EUGR were calculated at 36 and 40 weeks of GA, at discharge, and at 28 days. The concordance between the two definitions was estimated by Kappa coefficient. At 24–30 months, 803 infants were measured again. The agreement between the two definitions of EUGR was low. Both EUGR and not-EUGR groups were at lower centiles for weight, but at higher centiles for head circumference at 24–30 months than at birth. Longitudinal EUGR was associated with a poorer growth outcome for weight and height circumference than cross-sectional EUGR. No differences were observed for length. An agreed definition of EUGR is highly desirable in clinical practice to assess medical and nutritional interventions in preterm neonates. Based on the results of this study, we recommend the use of the longitudinal evaluation, that proved to better predict the auxological long-term outcome with respect to the cross-sectional one.

## 1. Introduction

Extrauterine Growth Restriction (EUGR) has often been reported in literature following preterm birth and birth with Very Low Birth Weight (VLBW, i.e., birthweight ≤ 1500 g). The condition of EUGR refers to an inadequate growth that occurs during hospitalization [1]. At discharge, preterm and VLBW infants often fail to gain weight according to expectations based on intrauterine growth charts; their weight is often at lower percentile with respect to birth and below the 10th percentile of expected growth [2,3]. The growth deficit can be observed for weight but also for head circumference and length [4].

EUGR is influenced by a number of factors. It can be partially explained by periods of inadequate nutrition, feeding intolerance (commonly present in preterm infants), and a range of mild to severe morbidities associated with preterm birth [5,6]. Prenatal and postnatal growth rates have been shown to be important for long-term outcomes [7,8,9,10,11,12,13,14,15,16]. There is increasing evidence that children who experienced a transient phase of preterm growth restraint develop a number of sequelae that are independent of whether the restraint occurred in utero (resulting in a small-for-gestational-age (SGA) infant), ex utero (preterm birth followed by poor neonatal growth), or in both phases [17]. It is still unclear to what extent EUGR affects long-term growth, the main reasons being controversial data from literature and the lack of a harmonized EUGR definition.

Current definitions can be classified in two groups: (1) cross-sectional, consisting of having a weight below the 10th centile (or another cut-off) at a given time (t), independently of birthweight; and (2) longitudinal, consisting of a weight loss of more than 1 (or 2) Standard Deviation (SD) between birth and a given t-time. The t-times proposed in literature are three: 36 weeks of GA, age at discharge, and 28 days of postnatal age [18]. 

The aim of this study was to investigate whether the EUGR (however defined) can predict the auxological outcome at 24–30 months of age, and to evaluate the agreement between cross-sectional and longitudinal definitions at the same t-time.

## 2. Methods

**Figure 1:**
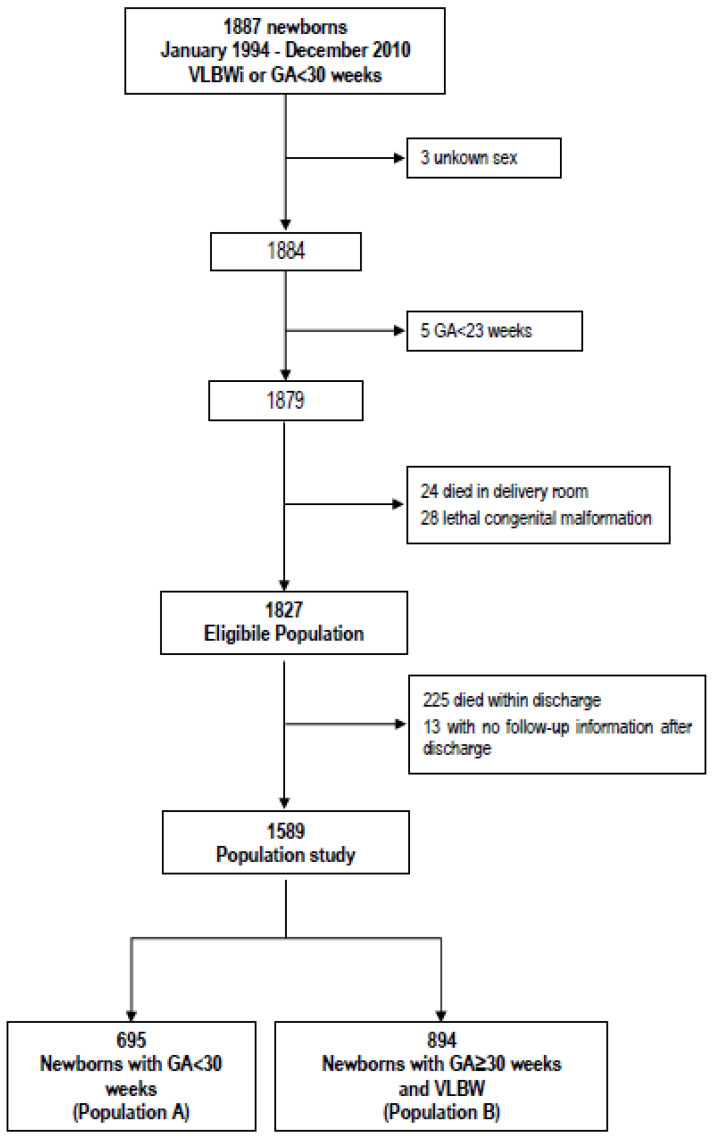
Inclusion criteria and definition of population study. GA: gestational-age; VLBWi: Very Low Birth Weight infants; VLBW: Very Low Birth Weight.

Clinical and growth data were collected for 1887 infants with GA < 30 weeks or weight ≤ 1500 g (according to the Vermont Oxford Network criteria [19]), born between 1994 and 2010 and admitted to the Neonatal Intensive Care Unit of the University of Turin, Italy. Exclusion criteria were major congenital malformations or genetic syndromes and death prior to discharge. In total, 1589 infants were included in the study. The inclusion threshold indicated by the Vermont Oxford Network (VON) and based on Gestational Age (GA) or on birthweight identifies two clinically different populations. The first one includes all neonates born with GA < 30 weeks (independently of their birth weight), while the second one includes neonates with GA ≥ 30 weeks only if VLBW (Figure 1). As a consequence, a higher percentage of major morbidities related to low GA was expected in the first population, whereas a higher proportion of females, twins (both physiological smaller), and SGA babies (by definition smaller) was expected in the second population. For this reason, the analysis considered separately the two different populations: (1) Population A (newborns with a GA < 30 weeks, n = 695) and (2) Population B (VLBW with GA ≥ 30 weeks, n = 894).

The weight was expressed in Standard Deviation Score (SDS or z-score) using the INeS charts [20] (at birth, and at the four t-times) and the CDC2000 charts (at 24–30 months) [21] as reference.

Neonates were defined asSGA or Large for GA (LGA) if their birthweight was below the 10th or above the 90th centile of the INeS charts, respectively.

EUGR was defined in the cross-sectional way if weight was below the 10th centile at a given t-time, whereas it was defined in the longitudinal way if the weight loss was more than one SD between birth and a given t-time. Forty weeks of GA was also considered in addition to the t-times typically considered in the literature (i.e., 36 weeks of GA, age at discharge, and 28 days of postnatal age), this being, conventionally, the average duration of a physiological pregnancy.

The agreement between cross-sectional and longitudinal EUGR definitions at the same t-time was estimated by Cohen’s kappa coefficient.

Among 1589 neonates, 803 babies (406 population A, 397 population B) were followed up until 24–30 months; their data were analyzed to assess the auxological outcome with a general linear model that considers as response the difference of weight-, length-, or head circumference-SDS between 24–30 months and birth (∆SDS), and as covariates the EUGR (defined on weight as above), the population (A or B), and the weight by GA (SGA or non-SGA).

## 3. Results

Table 1 reports the baseline characteristics of the two Populations (A and B). As expected, population B has a higher median GA and a higher proportion of singleton and females. The auxological variables at birth are, on average, higher in Population B than in Population A when the original values are considered. On the contrary, the mean SDSs are close to the expected values in population A (between −0.10 and −0.02, corresponding to 46th and 49th centile), and lower in Population B (between −1.29 and −1.09, corresponding to 10th and 14th centile). 

Table 2 shows major morbidities before discharge. GA was the main risk factor for severe morbidities.

Table 3 reports the number of EUGR-babies according to the two definitions (cross-sectional and longitudinal) at the different t-times. In Population A, the proportion of non-SGA infants with longitudinal EUGR was higher than that with cross-sectional EUGR, whereas in Population B an opposite tendency was observed. When considering both populations together, most SGA babies had cross-sectional EUGR; however, when EUGR is defined in a longitudinal way, the percentage of SGA babies with EUGR decreased. This was particularly evident in Population B. It should be noted that the agreement between cross-sectional and longitudinal definition of EUGR at the same t-time was low (−0.07 < Cohen’s kappa coefficient < +0.38).

Table 4 reports the raw means (SD) of auxological variables at 24–30 months of life. In average, the children are lighter and shorter and have e lower head circumference respect to the CDC-target population. 

The results of the general linear model estimate the effect of EUGR on growth, is reported in Table 5.

The LSmeans represents the estimate of the EUGR effect corrected for Population (A or B) and birth weight by GA (SGA or non-SGA).

Both EUGR and non-EUGR groups were, on average, around lower centiles for weight at 24–30 months than at birth (mean ∆SDS < 0). On the contrary, regarding the head circumference, both EUGR and non-EUGR groups were, on average, on higher centiles than at birth (mean ∆SDS > 0).

When EUGR was considered as longitudinal, the worsening of weight-SDS at 24–30 months was greater in EUGR than in non-EUGR group at all four times (p < 0.01). Similarly, the improvement of head circumference-SDS was higher in the non-EUGR than in the EUGR group. There were no differences between the EUGR and the non-EUGR in the cross-sectional evaluation, neither for weight nor for head circumference.

No differences were observed for length.

## 4. Discussion

In the literature, there is no consensus about the definition of EUGR. For this reason, the results of the available studies are difficult to compare. 

Regarding the cross-sectional definition, many studies available in the literature differ for population and time.

Applying the definition of EUGR as weight below the 10th centile at discharge, we estimated a prevalence of 74% of neonates with GA < 30 weeks, and 93% of neonates with GA ≥ 30 weeks and birthweight ≤ 1500 g. Our data are quite different with respect to the previous results described in literature; however, Clark et al. [1], Radmacher et al. [22], and Karagol et al. [23] analyzed significantly different populations. In particular, Clark et al. reported 28% of EUGR in a population of infants with birthweight < 2000 g and GA ≤ 34 weeks, Radmacher et al. [22] found a proportion of 59% in a population of newborns with birthweight < 750 g and GA < 29 weeks, and Karagol et al. reported 17% in population of non-SGA babies with birthweight between 750 g and 1250 g [23]. 

According to the definition of EUGR as weight below the 10th centile at 36 weeks of GA, we classified as EUGR 83% of infants with GA < 30 weeks, and 98% of newborns with GA≥30 weeks and birthweight ≤ 1500 g. Our results are in agreement with the data reported by Lemons et al. [24], who observed that 97% of VLBW newborns with GA ≤ 30 weeks presented EUGR. 

Applying a different definition of EUGR, characterized as weight at 28 days of postnatal life below the 10th centile, we found a prevalence of 53% of EUGR in infants with GA < 30 weeks, and 89% in newborns with GA ≥ 30 weeks and birthweight ≤ 1500 g. Our data agree with the results reported by Martin et al. [25], who estimated a EUGR proportion of 75% in a population of newborns with GA < 28 weeks. On the other hand, Karagol et al. [23] estimated a EUGR prevalence of only 26%, although in a population of non-SGA babies with birthweight between 750 g and 1250 g.

Regarding the longitudinal definition, in the literature, there are many studies that differ not only for population, but also for the choice of ∆SDS threshold. 

In our study, we defined the EUGR as the loss of 1SD between birth and discharge and 92% of newborns with GA < 30 weeks, and 55% of babies with GA ≥ 30 weeks and birthweight ≤ 1500 g was classified as EUGR. Franz et al. [26] defined EUGR as loss of 1SD from birth to discharge and estimated 50% of EUGR in a population of VLBW with GA < 26 weeks. 

Marks et al. [27], Shlomai et al. [2], and Roggero et al. [28] defined EUGR as loss of 2SD from birth to discharge. They applied it to a population of VLBW newborns [2,27] and to a population of VLBW and AGA newborns [28], observing 10.6%, 5.2%, and 13% of EUGR, respectively. 

Concerning the four times considered, each one of them presents advantages and disadvantages. The time of discharge depends on different criteria and often varies among the Neonatal Units and even among physicians of the same Unit. However, this time is often used because it is considered a proxy of the clinical stability of patient. The reliability of times of 36 and 40 weeks of GA depend on the gestational age at birth, and, when the GA at birth is too close to 36 or 40 weeks, the physiologic weight loss could be confused with EUGR. The time of 28 days of postnatal life does not depend on GA at birth and it is equidistant from birth for all neonates; however, the late preterm babies are likely to be discharged before this time.

Using different definitions of EUGR has several clinical implications. The cross-sectional way is an evaluation that does not consider what happened between birth and the t-time. The longitudinal assessment instead reflects changes from birth to a defined t-time. Additionally, for the longitudinal assessment, it is important to choose the right cut-off value to define EUGR (usually, weight loss of 1SD or 2SD) because the probability of exceeding it depends on birthweight: the lower the birthweight centile, the lower the probability to lose 1 or 2 SD. For this reason, and considering the large amount of SGA in our Neonatal Intensive Care Unit, we have chosen weight loss of 1SD as a cut-off of the longitudinal assessment.

The benefit of a harmonized definition of EUGR is of the use of this measure in clinical practice for identifying babies with high risk of long-term auxological or neurological negative outcomes [8,9,10,11,12,13,14,15,16].

The analysis of the auxological outcome at two years shows that, on average, our study population is lighter and shorter and has a lower head circumference with respect to the CDC-target population at the same age. This means that a delay in growth is experienced between birth and 24–30 months. Similar findings are reported in the literature [29,30,31,32]. 

When Population A (GA at birth < 30 weeks) and Population B (GA at birth ≥ 30 weeks and VLBW) were considered separately, centile crossing down was observed for all three auxological variables in Population A. In population B, weight centile crossing down (lower than in Population A) was observed; however, head centile crossing up and an almost length centile tracking was observed. This is probably due to GA, which is known to affect the postnatal growth more than other conditions [30,31].

The longitudinal definition of EUGR (contrary to the cross-sectional one) was predictive of growth at 24–30 months, showing that the EUGR babies have a higher delay of growth than non-EUGR ones. 

Regarding head circumference at 24–30 months, on average, it is of higher centiles than at birth. Additionally, in this case, only the longitudinal assessment of EUGR was predictive of the outcome, showing that, on average, the gain is higher in non-EUGR than in EUGR group. Based on the data reported in our study, we suggest to use the longitudinal definition of EUGR as a better predictor of the auxological long term-outcome. The t-time of choice in our clinical practice is “at discharge” due to the high concordance between operators in the discharge criteria.

The present study has limitations inherent to an observational study with retrospective collected data. For this reason, not all variables are available for all subjects at all the t-times considered. Moreover, even if the sample size is quite large, our study is monocentric. Since this is a single center study, the generalization of results to other centers is limited. On the other hand, the homogeneity of samples from a single center may strengthen the validity of the data. Future research should be designed as prospective, multi-center studies, in order to clarify the discrepancy in results across centers.

## 5. Conclusions

In conclusion, the low agreement between cross-sectional and longitudinal definitions of EUGR is likely due to the fact that they measure different aspects of the growth. For this reason, it is necessary to have a clear and harmonized definition of EUGR allowing to compare different clinical strategies and nutritional care of preterm infants during hospitalization in Neonatal Intensive Care Unit. This definition should be an indicator useful to clinicians in their everyday practice. Based on the results of this study, we recommend the use of the longitudinal evaluation, which proved to better predict the auxological long-term outcome with respect to the cross-sectional one. 

## Figures and Tables

**Table 1:** General characteristics of babies by population.

	Population A (N = 695)	Population B (N = 894)
Females, n (%)	342 (49.2)	492 (55.0)
Singleton, n (%)	527 (75.8)	588 (65.8)
Gestational Age (week), median (IQR)	28 (27–29)	31 (30–33)
Birth weight (g), mean (SD)	991 (255)	1251 (193)
Birth length (mm), mean (SD)	356 (30)	387 (22)
Head circumference (mm), mean (SD)	252 (19)	277 (14)
Birth weight (SDS), mean (SD)	−0.02 (0.946)	−1.29 (0.845)
Birth length (SDS), mean (SD)	−0.10 (0.928)	−1.09 (0.976)
Head circumference (SDS), mean (SD)	−0.06 (1.046)	−1.24 (1.013)
SGA, n (%)	76 (10.9)	459 (51.3)
LGA, n (%)	51 (7.3)	0 (0.0)
Antenatal steroids, n (%) *	534 (86.1)	647 (87.6)

* computed on 620 neonates of population A and 739 of population B. IQR: InterQuartile Range; SD: Standard Deviation; SDS: Standard Deviation Score; SGA: Small for gestational age; LGA: Large for gestational age.

**Table 2:** Major morbidities during hospital stay.

	Population A (N = 695)	Population B (N = 894)
Major morbidities, n (%)	621(89.4)	349(39.0)
Late sepsis, n (%)	120(17.3)	34 (1.9)
NEC, n (%)	18(2.6)	17(3.8)
ROP, n (%)	44(6.3)	2(0.2)
BPD, n (%)	24(3.5)	5(0.6)
RDS, n (%)	598(86.0)	299(33.5)
MV, n (%) *	568(83.5)	231(28.9)
HFOV, n (%) **	67(10.9)	10(1.3)
IVH, n (%)	43(7.4)	6(1.0)
PVL, n (%)	32(5.7)	19(3.2)
PDA, n (%)	292(42.0)	73(8.2)
Age at discharge in days		
median (min-max)	65 (24–137)	39 (17–90)

* computed on 680 neonates of population A and 800 of population B. ** computed on 617 neonates of population A and 767 of population B. NEC: necrotizing enterocolitis; ROP: retinopathy of prematurity; BPD: bronchopulmonary dysplasia; RDS: Respiratory Distress Syndrome; MV: Mechanical Ventilation; HFOV: High-frequency oscillatory ventilation; PVL: periventricular leukomalacia; IVH: intraventricular hemorrhage; PDA:patent ductus arteriosus.

**Table 3:** Infants with EUGR defined in cross-sectional and longitudinal way at the four different t-times.

EUGR Definition	Cross-sectional				Longitudinal			
	36 Weeks	40 Weeks	28 Days	Discharge	36 Weeks	40 Weeks	28 Days	Discharge
**Study population**								
non-SGA, n (%)	489/561 (87.2)	163/262 (62.2)	335/572 (58.6)	565/732 (77.2)	487/561 (86.8)	179/262 (68.3)	390/572 (68.2)	626/732 (85.5)
SGA, n (%)	340/340 (100.0)	159/167 (95.2)	250/250 (100.0)	331/332 (99.7)	109/340 (32.1)	90/167 (53.9)	62/250 (24.8)	145/332 (43.7)
**Population A**								
non-SGA, n (%)	268/331 (81.0)	107/170 (62.9)	168/358 (46.9)	318/447 (71.1)	312/331 (94.3)	135/170 (79.4)	260/358 (72.6)	414/447 (92.6)
SGA, n (%)	47/47 (100.0)	43/43 (100.0)	46/46 (100.0)	49/49 (100.0)	36/47 (76.6)	42/43 (97.7)	11/46 (23.9)	43/49 (87.8)
**Population B**								
non-SGA, n (%)	221/230 (96.1)	56/92 (60.9)	167/214 (78.0)	247/285 (86.7)	175/230 (76.1)	44/92 (47.8)	130/214 (60.8)	212/285 (74.4)
SGA, n (%)	293/293 (100.0)	116/124 (93.6)	204/204 (100.0)	282/283 (99.7)	73/293 (24.9)	48/124 (38.7)	51/204 (25.0)	102/283 (36.0)

**Table 4:** Raw means of auxological variables at 24–30 months of life.

	Study Population	Population A	Population B
Weight (g), mean (SD)	11,146 (1578)	11,261 (1724)	11,029 (1406)
Length (mm), mean (SD)	853 (39)	853 (39)	853 (40)
Head circumference (mm), mean (SD)	477 (18)	477 (19)	477 (17)
Weight (SDS), mean (SD)	−1.43 (1.373)	−1.39 (1.492)	−1.47 (1.240)
Length (SDS), mean (SD)	−0.94 (1.060)	−1.01 (1.057)	−0.86 (1.059)
Head circumference (SDS), mean (SD)	−0.41 (1.163)	−0.43 (1.231)	−0.39 (1.088)

**Table 5:** **∆**SDS (SDS 24–30 months –SDS at birth) LS means ± Standard Error in EUGR and non-EUGR group. EUGR was defined using weight in cross-sectional and longitudinal way at the four t-times.

TRAIT	EVALUATION	t	EUGR			
			No	Yes	No vs. Yes	p
**WEIGHT**	**Cross-** **Sectional**	36 weeks of GA	−0.32 ± 0.192	−0.67 ± 0.056	+0.36 ± 0.201	0.077
		40 weeks of GA	−0.57 ± 0.153	−0.86 ± 0.086	+0.29 ± 0.180	0.110
		28 days	−0.95 ± 0.113	−0.71 ± 0.065	−0.24 ± 0.136	0.076
		discharge	−0.92 ± 0.121	−0.65 ± 0.051	−0.27 ± 0.133	**0.043**
	**Longitudinal**	36 weeks of GA	−0.23 ± 0.112	−0.82 ± 0.067	+0.59 ± 0.141	**<0.001**
		40 weeks of GA	−0.47 ± 0.130	−0.95 ± 0.093	+0.48 ± 0.166	**0.004**
		28 days	−0.58 ± 0.087	−0.93 ± 0.074	+0.36 ± 0.120	**0.003**
		discharge	−0.38 ± 0.099	−0.80 ± 0.055	+0.42 ± 0.118	**<0.001**
**LENGTH**	**Cross-** **Sectional**	36 weeks of GA	−0.09 ± 0.181	−0.14 ± 0.050	0.05 ± 0.189	0.772
		40 weeks of GA	−0.30 ± 0.129	−0.27 ± 0.073	−0.02 ± 0.152	0.872
		28 days	−0.46 ± 0.103	−0.19 ± 0.060	−0.27 ± 0.125	**0.030**
		discharge	−0.61 ± 0.108	−0.16 ± 0.046	−0.45 ± 0.120	**<0.001**
	**Longitudinal**	36 weeks of GA	+0.02 ± 0.100	−0.21 ± 0.061	+0.23 ± 0.126	0.072
		40 weeks of GA	−0.11 ± 0.107	−0.38 ± 0.079	+0.27 ± 0.137	0.053
		28 days	−0.18 ± 0.078	−0.33 ± 0.069	+0.15 ± 0.109	0.180
		discharge	−0.11 ± 0.089	−0.29 ± 0.051	+0.18 ± 0.107	0.094
**HEAD CIRCUMFERENCE**	**Cross-** **Sectional**	36 weeks of GA	+0.31 ± 0.159	+0.33 ± 0.046	−0.02 ± 0.167	0.924
		40 weeks of GA	+0.20 ± 0.126	+0.22 ± 0.071	−0.01 ± 0.148	0.939
		28 days	−0.01 ± 0.094	+0.22 ± 0.053	−0.23 ± 0.113	**0.045**
		discharge	+0.07 ± 0.104	+0.32 ± 0.043	−0.25 ± 0.114	**0.029**
	**Longitudinal**	36 weeks of GA	+0.64 ± 0.092	+0.20 ± 0.055	+0.44 ± 0.115	**<0.001**
		40 weeks of GA	+0.34 ± 0.108	+0.15 ± 0.077	+0.20 ± 0.138	0.156
		28 days	+0.33 ± 0.072	+0.03 ± 0.061	+0.30 ± 0.100	**0.003**
		nidischarge	+0.52 ± 0.084	+0.20 ± 0.047	+0.32 ± 0.100	**0.001**

Note: p values in bold are those considered significant.
